# Comparison of flanged and unflanged acetabular cup design: An experimental study using ceramic and cadaveric acetabuli

**DOI:** 10.3109/17453674.2010.519167

**Published:** 2010-10-08

**Authors:** Mette Ørskov, Saba Abdulghani, Ian McCarthy, Kjeld Søballe, Gunnar Flivik

**Affiliations:** ^1^Biomaterials and Biomechanics Laboratory, Department of Orthopedics, Lund University and Skåne University Hospital, Lund, Sweden; ^2^Department of Orthopaedics, Ribe County Hospital, Esbjerg; ^3^Department of Orthopaedics, Aarhus University Hospital, Denmark

## Abstract

**Background and purpose:**

Adequate depth of cement penetration and cement mantle thickness is important for the durability of cemented cups. A flanged cup, as opposed to unflanged, has been suggested to give a more uniform cement mantle and superior cement pressurization, thus improving the depth of cement penetration. This hypothesis was tested experimentally.

**Materials and methods:**

The same cup design with and without flange (both without cement spacers) was investigated regarding intraacetabular pressure, cement mantle thickness, and depth of cement penetration. With machine control, the cups were inserted into open-pore ceramic acetabular models (10 flanged, 10 unflanged) and into paired cadaver acetabuli (10 flanged, 10 unflanged) with prior pressurization of the cement.

**Results:**

No differences in intraacetabular pressures during cup insertion were found, but unflanged cups tended to migrate more towards the acetabular pole. Flanged cups resulted in thicker cement mantles because of less bottoming out, whereas no differences in cement penetration into the bone were observed.

**Interpretation:**

Flanged cups do not generate higher cementation pressure or better cement penetration than unflanged cups. A possible advantage of the flange, however, may be to protect the cup from bottoming out, and there is possibly better closure of the periphery around the cup, sealing off the cement-bone interface.

The main cause of aseptic loosening is inadequate surgical techniques and inferior prosthetic implants (Herberts and Malchau 2000). Sufficient cement penetration (3–5 mm) into cancellous bone and prevention of bottoming out of the cup, as seen from a uniform cement mantle that is at least 2 mm thick (i.e. cement penetration excluded), have been said to be crucial for cup fixation (Huiskes and Slooff 1981, Noble and Swarts 1983, Schmalzried et al. 1993, Mjöberg 1994, Ranawat et al. 1997, Lichtinger and Muller 1998). A clean bony surface with partly exposed cancellous bone together with cement pressurization before prosthetic implantation improves the depth of cement penetration, thus creating a stronger cement-bone interface (Krause et al. 1982, Rey, Jr. et al. 1987, Mann et al. 1997, Flivik et al. 2006, Abdulghani et al. 2007).

Absence of postoperative demarcation at the acetabular cement-bone interface has been related to a reduced risk of aseptic cup loosening (Ranawat et al. 1995, Garcia-Cimbrelo et al. 1997, Ritter et al. 1999, Flivik et al. 2005). The use of a flanged polyethylene cup has demonstrated both less postoperative demarcation at the above interface (Hodgkinson et al. 1993) and less loosening (Garellick et al. 2000). This may be due to its ability to increase cement pressurization at the time of implantation and thereby the depth of cement penetration, though conflicting experimental findings have been reported (Oh et al. 1985, Shelley and Wroblewski 1988, Parsch et al. 2004, Lankester et al. 2007). The previous studies addressing the use of flanged cups have all had cups inserted without prior pressurization of cement, and only Parsch et al. (2004) implanted the cup into a porous material (cadaveric bone).

Accordingly, we decided to compare the intraacetabular pressures, cement mantle thickness, and depth of cement penetration obtained using flanged and unflanged cups inserted in an open-pore ceramic acetabular model as well as in paired cadaveric acetabuli, using pressurization of the cement before implantation.

## Material and methods

### Ceramic study

**Figure 1.**
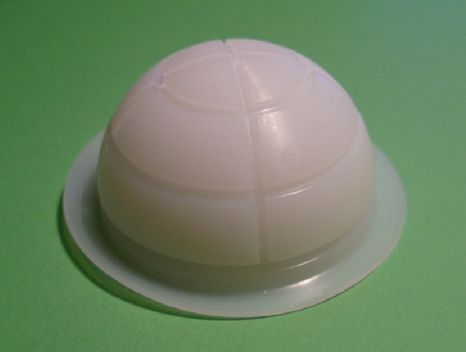
The Opera cup (with flange).

20 ceramic acetabular models with a diameter of 49 mm were produced from Sivex ceramic foam filter plates (filter grade 80, cell size 600–700 microns; Pyrotek SA, Sierre, Switzerland). 2 custom-made pressure sensors (modified Entran, EPB; Entran Sensors and Electronics, Garston, UK) with a diameter of 3.6 mm and a 100-mm shaft were inserted through a standardized drill hole located at the acetabular pole, and 2.5 cm from the rim, respectively (holes were drilled using a specially designed drill guide). The tip of each sensor was covered with tape to protect it from polymer-induced damage, and it was made level with the acetabular surface. 20 cross-linked-polyethylene XLPE Opera cups (Smith and Nephew, Andover, MA) with a 43-mm outer diameter (flange excluded), a 28-mm inner diameter, and no orientation wire were used (Figure 1). 10 cups had the flange completely cut off (unflanged sockets) and the remaining 10 had the flange trimmed (flanged prostheses) to fit just on top of the acetabular model. To protect the brittle ceramic rim, the flange was not trimmed to fit inside the reamed hemisphere. Every cup was inserted with 40 g of prechilled (5°C) Refobacin-Palacos R cement (Biomet, Warsaw, IN) using an Instron 851120 materials testing machine (Instron Corporation, Norwood, MA). The temperature of the room was kept a 20°C, and the cement was removed from the refrigerator just before vacuum mixing in the Optivac mixing system (Biomet Cementing Technologies AB, Sjöbo, Sweden). 2.5 min after the onset of mixing, cement was applied in the acetabular model, and pressurized with 80 N for 1.5 min using a conventional pressurizer (Smith and Nephew), which was fitted into the Instron machine. 5 min after the onset of mixing, the cup was inserted, position-controlled by the use of a femoral head and a specially designed device to avoid tilting of the cup during introduction. Thereafter, the cup was held in place with force control (25 N) until the cement had cured. The resulting forces, pressures, and cup displacements were recorded continuously every 0.02 seconds during cementation using Spider8 software (HBN Inc., Marlborough, MA). After the cementing procedures, all samples were cut longitudinally along the center of the cup with an electric saw, and digitized using an HP scanjet 4470c digital flatbed scanner (1,200 dpi) to enable inspection of the cement mantle and penetration depth (Abdulghani et al. 2007).

### Cadaver study

10 human cadaver pelvises embalmed in 5% (v/v) formalin, 45% ethanol, 27% glycerine, and 5% glyoxide-glutaraldehyde were obtained from the Anatomical Institute, Aarhus University, Aarhus, Denmark. All pelvises were from male donors (median age 83 (65–102) years) without any previous hip surgery or signs of osteoarthritis. The left and right acetabulum was randomly allocated to receive a cup either with or without a flange. The flange was either trimmed to fit inside the acetabulum (flanged cup) or cut off (unflanged cup). All acetabuli were over-reamed according to the manufacturer's recommendations using a conventional reamer, to provide a final cement mantle between 2.5 and 3.5 mm, depending on the size of the last reamer, and the most suitable cup size (40, 43, 47, 50, or 53 mm). Every acetabulum in a pair was equally over-reamed, and the same cup size was inserted on both sides. During reaming, the aim was to remove at least 75% of the subchondral bone plate area in order to maximize the possibility of cement penetration by exposing cancellous bone (Flivik et al. 2006). 9 anchorage holes 6 mm in diameter and 6 mm deep were drilled with a standardized distribution, i.e. 1 anchorage hole in os pubis and os ischii, respectively, and the remaining 7 holes drilled in os ilium. All acetabular preparations were done by an experienced hip surgeon (GF).

**Figure 2.**
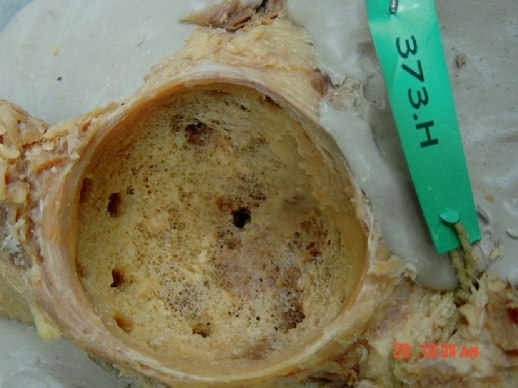
Prepared cadaveric acetabular bone bed with the cancellous bone exposed.

Afterwards, every acetabular bone was potted into Vel-Mix Stone (Kerr Italia S.p.A., Scafati, Italy) to ensure horizontal alignment of the acetabular opening during further handling. Finally, 2 additional channels for the later application of pressure sensors were drilled at the pole and 10 mm from the iliac rim (opposite the transverse ligament, using a specially designed device). All acetabuli were then cleaned with pulse lavage, and before cementation the acetabular bone bed was dried with gauze (Figure 2). Subsequently, the previously used pressure sensors were inserted (the sensor tips were again leveled with the cancellous bone surface), and a flanged or unflanged cup was implanted using 40 g of prechilled (5°C) Refobacin-Palacos R cement under identical conditions with pressurization of the cement before insertion as described for the ceramic study. After cementation, every cadaveric bone pair was reversely aligned and CT-scanned in the coronal plane using a Philips Mx8000 IDT 16 CT scanner (Philips Medical Systems, Andover, MA) with the following settings: 120 kV, 158 mA, and a 0.8 mm slice thickness to enable estimation of the total cement volume and penetration depth. The bones were stored in a cold room between cadaveric handling.

### Data management

#### Intraacetabular pressures and cup displacements in ceramic and cadaveric acetabuli

Insertion forces and intraacetabular pressure measurements were obtained during position-controlled cup insertion within the last 3 mm before the final cup position. Resulting intraacetabular pressures and cup displacements were also assessed during force-controlled pressurization. Area under the curve (AUC) was computed for every insertion force and pressure measurement (with use of the trapezoid rule), and subsequently the calculated value was divided by the observed time period (Flivik et al. 2004). Cup displacements obtained under a constant force were evaluated for 45 and 150 seconds, with a negative number indicating cup migration towards the acetabular pole.

#### Cement mantle thickness, penetration depth, and areas in ceramic

**Figure 3.**
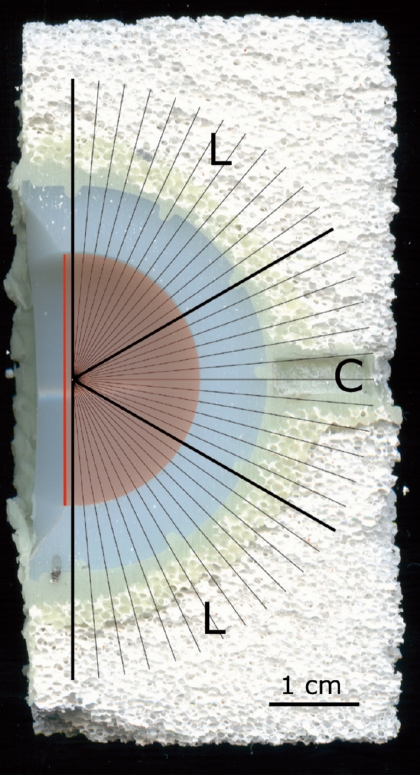
The template with test lines placed on a sample of ceramic. Note the close contact between the unflanged cup and the ceramic. Lateral segments are labeled L, and the central segment C.

A hemisphere template was created in Adobe Photoshop 7.0 to divide the acetabulum into three 60° segments (2 laterals and 1 central). Each segment was divided into 12 subregions by adding a radial test line for every 5 degrees (Figure 3). Cement mantle thickness and penetration depth were measured along every test line, with the exception of the central zone, where only 6 measurements were performed in the lateral part of the region to avoid uncertainty caused by the pole pressure sensor channel. Accordingly, the median mantle thickness and penetration depth could be calculated. The lateral and central mantle and penetration area for every 5 degrees were also estimated. Penetration was defined to begin at the base of a proximal penetration sprout, and to end at the most distal point of cement along a radial test line. All measurements were performed with ImageJ software (ImageJ 1.31i, W. Rasband, NIH, MD).

#### Total cement volume and penetration in cadaveric acetabuli

**Figure 4.**
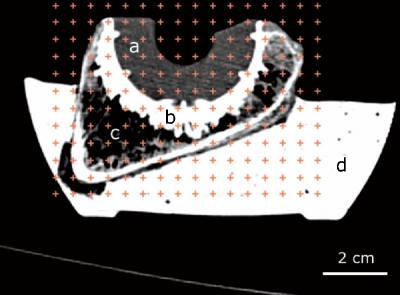
The counting grid placed on a CT image of cadaveric bone. Opera cup (a), cement (b), cadaveric bone (c), and Vel-Mix stone (d).

The total cement volume (mantle thickness plus penetration depth) was estimated using Cavalieri's direct estimator (Gundersen et al. 1988). Basically, a grid containing points covering a known area was created (Adobe Photoshop 7.0); then, in the total upper-right corner the points overlaying the cement were counted in every twelvth CT slide (Figure 4). The starting point was random, and 12–15 slides were analyzed in a sample using an equal number of slides for the other half of the bone pair. All analyses were performed blind. When estimating cement penetration, a medial and a lateral anchorage hole were localized for every sample on the CT sections, and the images giving the most prominent diameter were chosen. In the opposite cadaveric bone pair, the corresponding anchorage hole was selected. The diameter of each anchorage hole chosen (i.e. the diameter of the drill hole plus penetration at both sides of the hole) was measured 3 times at its thickest location (with ImageJ). Penetration was subsequently calculated as half of the difference between the median measured diameter and the known size of the drill hole (6 mm). All analyses were performed blind.

### Statistics

A minimum sample size of 8 in the cadaveric study was calculated to achieve sufficient power (> 80%) based on published pressure variation data (Parsch et al. 2004) and assuming a 100 mmHg difference in the obtained median pole pressures between flanged and unflanged cups. STATA version 7 (StataCorp LP, College Station, TX) was used for all statistical analyses, with p-values of ≤ 0.05 being regarded as significant. Ceramic groups were compared with the Mann-Whitney U test, whereas cadaveric groups were compared using the Wilcoxon signed rank test. Data are presented as median values with the 95% confidence interval in brackets, unless otherwise stated.

## Results

### Intraacetabular pressures, forces, and cup displacement in ceramic

Forces, pressures, and cup displacements were measured for 3 flanged cups and 3 unflanged cups (Table 1). During position-controlled insertion of the cup, no statistically significant differences were obtained between the insertion forces or intraacetabular pressures when comparing flanged an unflanged cups. In addition, both types of cup produced similar intraacetabular pressures (flanged, p = 0.5; unflanged, p = 0.1), i.e. pole and rim pressures were similar. However, during force-controlled pressurization the unflanged cups showed deeper displacement in the direction toward the acetabular pole (p = 0.05) and produced a higher pole pressure than the flanged cups did (p = 0.05) (Table 1). In addition, the intraacetabular pressures turned out to be unevenly distributed only in the unflanged cup, with a higher pressure obtained at the pole.

**Table 1.** Intraacetabular pressures in ceramic. Values are median (95% confidence interval)

	Flanged	Unflanged	p-value
Position-controlled cup insertion			
Force applied (N)	68 (65–74)	57 (43–83)	0.5
Pole pressure (mmHg)	353 (281–356)	402 (298–568)	0.3
Rim pressure (mmHg)	283 (282–292)	252 (189–331)	0.5
Force-controlled pressurization			
Cup displacement (mm) ^**a**^	-0.1 (-0.2–0.0)	-0.2 (-0.4 – -0.2)	0.05
Pole pressure (mmHg)	86 (0.0–108)	140 (114–160)	0.05
Rim pressure (mmHg)	76 (8–85)	25 (24–55) **^b^**	0.5

**^a^** Negative displacement indicates cup migration toward the acetabular pole.

**^b^** p < 0.05 for pole vs. rim pressures.

### Cement mantle thickness, penetration depth, and areas in ceramic

Mantle thickness, penetration depth, and the respective areas were measured in 10 flanged and 10 unflanged samples (Table 2). The central and the lateral cement mantle thicknesses were statistically significantly thicker with a flanged cup than with an unflanged cup, and also the central and lateral cement mantle area was significantly larger when a flanged cup was used. The mantle thickness and area were equally distributed for the flanged cup (p = 0.1 for thickness and p = 0.2 for area), and also for the unflanged cup (p = 0.8 and p = 0.3). The depth of cement penetration and cement penetration area were similar for the two cups (Table 2). Both cups were observed to have deeper penetration centrally than laterally (p = 0.002 for the flanged cups and p = 0.003 for the unflanged cups), and we obtained similar results for the penetration area (p = 0.003 and p < 0.001).

**Table 2.** Thickness of cement mantle, depth of penetration, and area of penetration per 5° sector in ceramic. Values are median (95% confidence interval)

	Flanged	Unflanged	p-value
Cement mantle			
Lateral thickness (mm)	2.4 (1.7–3.8)	1.5 (0.9–2.0)	0.002
Central thickness (mm)	3.3 (2.0–4.2)	1.6 (0.6–2.1)	<0.001
Lateral area (mm^2^/5°)	4.6 (3.2–7.1)	2.9 (2.1–4.1)	<0.001
Central area (mm^2^/5°)	5.8 (3.7–7.7)	2.4 (1.0–4.1)	<0.001
Cement penetration			
Lateral depth (mm)	3.6 (3.2–4.0)	3.8 (3.2–4.2)	0.5
Central depth (mm)	4.2 (4.6–5.3) **^a^**	4.7 (3.2–5.9) **^a^**	0.6
Lateral area (mm^2^/5°)	9.2 (7.9–9.5)	8.8 (8.3–9.6)	0.2
Central area (mm^2^/5°)	10.2 (8.9–12.1) ^**a**^	10.4 (9.1–11.6) **^b^**	0.4

**^a^** p <0.01 for lateral vs. central measurements.

**^b^** p <0.001 for lateral vs. central measurements.

### Intraacetabular pressures, force, and cup displacement in cadaveric acetabuli

During position-controlled cup insertion, forces and pressures were collected for 10 paired samples (Table 3). The insertion forces were similar for flanged and unflanged cups. Unflanged cups produced higher intraacetabular pole pressures than flanged cups, but both types of cup produced uneven intraacetabular pressures with highest pressures obtained at the pole (p = 0.02 for flanged cups and p = 0.005 for unflanged cups). During force-controlled pressurization, cup displacements were collected for 8 paired samples and pressures were obtained for 9 paired samples (Table 3). Again, the unflanged cups migrated more towards the acetabular pole and produced higher pole pressures than the flanged cups did. In contrast to the unflanged cups, only the flanged cups produced uniform pressures when comparing pole pressures with rim pressures.

**Table 3.** Intraacetabular pressures in cadaveric bone. Values are median (95% confidence interval)

	Flanged	Unflanged	p-value
Position-controlled cup insertion			
Force applied (N)	90 (12–196)	75 (0–138)	0.8
Pole pressure (mmHg)	218 (7–524)	470 (25–1,656)	0.04
Rim pressure (mmHg)	156 (3–457) ^**a**^	196 (2–596) ^**a**^	0.4
Force-controlled pressurization			
Cup displacement (mm) **^b^**	-0.1 (-0.4 – 0.1)	-1.0 (-1.7–0.1)	0.01
Pole pressure (mmHg)	12 (3–100)	130 (21–190)	0.008
Rim pressure (mmHg)	17 (3–80)	23 (4–123) **^a^**	0.6

**^a^** p < 0.05 for pole vs. rim pressures.

**^b^** Negative displacement indicates cup migration toward the acetabular pole.

### Total cement volume and penetration depth in cadaveric acetabuli

Total cement volume and penetration were measured in 10 paired samples. Flanged cups were found to be enclosed by more cement than unflanged cups (70 (58–76) cm3 as opposed to 57 (46–76) cm3; p = 0.005), whereas the cement penetration was similar between the two cup types (0.92 (0.30–2.61) mm as opposed to 0.99 (0.21–2.34) mm; p = 0.9).

## Discussion

Increased cement penetration into the acetabular bone improves cup stability (Flivik et al. 2005). However, many factors influence cement penetration including magnitude and duration of the force applied, properties of the bone cement used, amount of bone bleeding, anatomy, porosity, and (not least) preparation of the acetabular bone (Noble and Swarts 1983, Juliusson et al. 1994, Graham et al. 2003, Hogan et al. 2005, Flivik et al. 2006).

The possible advantage of a flanged cup has mainly been related to its hypothesized ability to increase cement pressurization at the time of implantation, thus improving cement penetration (Oh et al. 1985, Shelley and Wroblewski 1988). When we inserted a flanged and an unflanged cup in a position-controlled manner using equivalent forces, the intraacetabular pressures were similar between both types of cup inserted in ceramic acetabuli, and both types produced equal intraacetabular pressures as well. When the cups were pressurized further (using force-control), the unflanged cups migrated more towards the acetabular pole than the flanged cups did when inserted both in ceramic and paired cadaveric acetabuli, despite the minimal application of force (25 N). However, the migration susceptibility observed for the unflanged cup was most certainly increased further due to the lack of cement spacers in this specific cup.

It has been suggested that there may be a correlation between the use of a flanged cup and a lower incidence of bottoming out (Oh et al. 1985). We know of no consistent classification concerning bottoming out. Using a tentative definition, of cement mantle thickness less than 1 mm along any of the 29 test lines (in the ceramic study), 9 of 10 unflanged cups, and just 2 of 10 flanged sockets showed bottoming out (p = 0.002). Close contact between polyethylene and bone has been related to reduced cup longevity (Wroblewski et al. 1987). It is thus tempting to suggest that the reduced cement mantle thickness observed in the unflanged cup experiments may reduce cup durability, but again the lack of cement spacers probably influenced these results and highlights the importance of cement spacers in cup designs without a flange. It should also be considered that a flanged cup may also risk an eccentric cement mantle (Sandhu et al. 2006), and care is needed when adjusting the flange to the particular acetabulum to avoid this.

The porosity and preparation of the acetabular bone bed is related to the degree of interdigitation of cement, and removal of the subchondral bone plate has been observed to improve the cement-bone interface and to lower the interfacial stresses without impairing prosthetic stability (Volz and Wilson 1977, Sutherland et al. 2000, Flivik et al. 2006). We know that all sockets inserted in both ceramic and cadaveric bone were implanted under good conditions, due to a dry acetabulum without any blood or bone-marrow to disrupt cement penetration (Krause et al. 1982, Ranawat et al. 1995). However, all prostheses were inserted under the same conditions, and the errors are therefore systematic. To adjust for the improved conditions regarding a dry acetabulum, we pressurized cement and prostheses with less force than is usually applied in the clinic.

The overall amount of cement penetration was higher in the ceramic study than in cadaveric bone. The reason may lie in the larger pore diameters and a completely open porous structure in ceramic. According to Poiseuille's law (R = 8 ηL × (π r4) – 1) where R denotes flow resistance, η viscosity, and L the length of the pores with radius r (Alkafeef et al. 2006). Larger pore diameters give less flow resistance, thereby facilitating higher penetration. The deeper central penetration observed in the ceramic study can be explained by the higher pressure gradient at this location. In fact, this is confirmed by the second part of Poiseuille's law (ƒ = ΔP × R – 1), in which the flow (ƒ) of the liquid is governed by the pressure gradient (ΔP) and the flow resistance (R).

We found no differences in depth of cement penetration between the cups that were tested when inserted in either ceramic or paired cadaveric acetabuli, which confirms earlier findings that cement penetration occurs mainly during cement pressurization before cup insertion (Abdulghani et al. 2007). Thus, when it is time to insert the cup the cement might simply be too viscous to permit further penetration, even with a flanged cup design. It seems as if the flanged cup reduces cement leakage during insertion—at least when investigating a cup design lacking cement spacers—and the increased cement volume we observed around the flanged cup in the cadaver study was most certainly caused by a thicker cement mantle. In most studies, however, no differentiation has been made between depth of cement penetration and cement mantle thickness, and the total is usually referred to as the cement mantle. If possible, both depth of cement penetration and cement mantle thickness should be considered.

In conclusion, we argue that as flanged cups do not appear to generate higher cement pressure or induce an increased degree of cement penetration, this is not the reason for the superior clinical outcome reported for some flanged cups (Hodgkinson et al. 1993, Garellick et al. 2000). Flanged cups have, however, been associated with less postoperative demarcation in the important cement-bone periphery around cups (Hodgkinson et al. 1993). A demarcation in this interface can represent an exposed surface where joint fluid and wear particles can act and start to fuel the aseptic loosening process, which is seen as a progressive radiolucent line (Schmalzried et al. 1992, Robertsson et al. 1997, Aspenberg and Van der Vis 1998, Van der Vis et al. 1998, McEvoy et al. 2002, Lankester et al. 2007). We suggest that apart from preventing the cup from botttoming out, the only advantage with a flange as regards longevity of the cup is its improved ability to close the periphery around the cup, and thereby to protect the cement-bone interface. The exact closure and fit of the flange to the acetabular rim is dependent on what shape the flange has and how well it is trimmed to fit by the surgeon.
